# Mediating Role of CMV Infection and GrimAge in the Association of Education With Health Outcomes in Cancer Survivors

**DOI:** 10.1093/geroni/igaf122.3205

**Published:** 2025-12-31

**Authors:** Shuo Wang, Weihua Guan, Heather Nelson, Bharat Thyagarajan, Susan Everson-Rose, Anne Blaes, Anna Prizment

**Affiliations:** University of Minnesota, Minneapolis, Minnesota, United States; University of Minnesota, Minneapolis, Minnesota, United States; University of Minnesota, Minneapolis, Minnesota, United States; University of Minnesota, Minneapolis, Minnesota, United States; University of Minnesota, Minneapolis, Minnesota, United States; University of Minnesota, Minneapolis, Minnesota, United States; University of Minnesota, Minneapolis, Minnesota, United States

## Abstract

Cancer survivors with lower education experience a disproportionate burden of health outcomes. These health disparities may be partially explained by cytomegalovirus (CMV) infection and aging biomarkers. In the Health and Retirement Study (HRS) – a nationally representative cohort of older adults, we examined whether CMV infection and GrimAge, an epigenetic clock, explained the associations of education (< high school vs. ≥high school) with comorbidity index and muscle weakness. HRS measured CMV Immunoglobulin G antibody levels in blood samples from 1,337 cancer survivors in 2016 (mean age=72 years, 54% female, 16% Non-white). A comorbidity index was constructed using five conditions (hypertension, lung disease, cardiac disorders, stroke, diabetes), and muscle weakness was assessed using grip strength in the dominant hand for these survivors. Additionally, GrimAge was calculated for 574 of the survivors. We calculated age acceleration for GrimAge as residuals after regressing GrimAge on chronological age to capture its effect independent of age (GrimAge_r). All analyses were adjusted for survey weights to generate nationally representative estimates. In the structural equation model adjusted for age, sex, race, BMI, and smoking, CMV infection explained 10.2% (p = 0.03) of the association between education and comorbidity index (total effect=0.30, p < 0.01). GrimAge_r explained 18.9% (p = 0.01) of the association between education and comorbidity index (total effect=0.41, p < 0.01). Neither CMV infection nor GrimAge_r mediated the association between education and muscle weakness; however, the sample size was limited. Our findings suggest that CMV infection and epigenetic clocks may mediate the association between education and health outcomes in cancer survivors.

